# Adherence to Mediterranean Diet: A Population-Based Longitudinal Cohort Study

**DOI:** 10.3390/nu15081844

**Published:** 2023-04-12

**Authors:** Elisa Mattavelli, Elena Olmastroni, Manuela Casula, Liliana Grigore, Fabio Pellegatta, Andrea Baragetti, Paolo Magni, Alberico L. Catapano

**Affiliations:** 1 Department of Pharmacological and Biomolecular Sciences, Università degli Studi di Milano, 20133 Milan, Italy; elisa.mattavelli@unimi.it (E.M.); andrea.baragetti@unimi.it (A.B.); 2 MultiMedica IRCCS, Sesto S. Giovanni, 20099 Milan, Italy; elena.olmastroni@unimi.it (E.O.); manuela.casula@unimi.it (M.C.); grigore.centroatero@gmail.com (L.G.); fabio.pellegatta@guest.unimi.it (F.P.); alberico.catapano@multimedica.it (A.L.C.); 3 Epidemiology and Preventive Pharmacology Service (SEFAP), Department of Pharmacological and Biomolecular Sciences, Università degli Studi di Milano, 20133 Milan, Italy

**Keywords:** mediterranean diet adherence, cardiovascular disease, metabolic disease, longitudinal study

## Abstract

Adherence to the Mediterranean diet (MedDiet) is recommended for cardiovascular disease prevention. However, recent epidemiological studies report a shift toward lower adherence to MedDiet. We have conducted a prospective cohort study to evaluate changes in individual determinants of MedDiet adherence over time. Clinical information and MedDiet adherence score (MEDAS) were collected in 711 subjects (mean age 68 ± 10 years; 42% males), enrolled in the PLIC study (Progression of Intimal Atherosclerotic Lesions in Carotid arteries), during two visits conducted, on average, 4.5 years apart. MEDAS score worsening and improvements (absolute change, ΔMEDAS) and the variation in the proportion of subjects reporting to meet each MEDAS criteria were assessed. Overall, 34% of the subjects improved their MedDiet adherence (ΔMEDAS: +1.87 ± 1.13), by consuming more olive oil, legumes and fish and use of dishes seasoned with sofrito and 48% subjects worsened their MedDiet adherence (ΔMEDAS: −2.02 ± 1.14) by consuming less fruit, legumes, fish and nuts, with higher rates of worsening in women and subjects aged 50–65 years. Subjects who improved the score were more obese, had higher plasma glucose levels, and metabolic syndrome at the basal visit. In summary, we report an overall decrease in MedDiet adherence, evaluated during a timeframe heavily affected by the COVID-19 pandemic, underlining the need for better dietary interventions.

## 1. Introduction

The traditional Mediterranean diet (MedDiet) is one of the most well-known and well-researched dietary patterns worldwide. As it was defined based on the traditional dietary pattern followed by the inhabitants of the Mediterranean region, it encompasses minimally processed food consumption such as high intake of plant foods (fresh and seasonal fruits and vegetables, legumes and nuts) and a moderate to low intake of foods of animal origin, giving preference to fish and poultry over others. Furthermore, MedDiet restricts the consumption of simple sugars, and favors the consumption of olive oil, as the main dietary source of fats [1,2].

Since the 1960s, the MedDiet has been extensively studied to understand its role in the prevention of chronic and/or degenerative diseases, cognitive decline, metabolic syndrome, cardiovascular diseases, and cancer [3].

MedDiet combines the synergistic effects of individual food components including healthy sources of fat, starch, proteins, fiber, vitamins and minerals and several bioactive compounds such as polyphenols and phytosterols with potentially beneficial health-related effects [4].

Indeed, dietary modifications toward higher MedDiet adherence have been related to decreasing overall as well as cardiovascular morbidity and mortality [5,6].

The landmark randomized primary prevention trial, the PREDIMED study, showed that the MeDiet provides long-term high benefits on CVD compared with a low-fat diet [7]. Subsequent meta-analyses have confirmed that increasing adherence to the Mediterranean diet reduces mortality from all causes [8,9], and this was coherently observed both in in Mediterranean and in non-Mediterranean areas, and both in stud-ies with shorter (for <7 years) and longer (≥7 years) duration.

Due to MedDiet protective effects on non-communicable disease incidence and the better adherence maintened over time, prevention guidelines support the Mediterranean or similar dietary patterns (harmonized by geographical location features [10]) as the best dietary approach for the management and mitigation of non-communicable disease risk factors [11], and many intervention trials have been conducted to improve adherence to MedDiet through individual visits and personalized dietary advice [12].

Despite these recommendations, large dietary surveys report a progressive shift from MedDiet toward a more Westernized diet [13]. A concomitant increase in non-communicable disease incidence [14] has also been reported, mainly due to increased cardiovascular disease incidence, which are in turn largely promoted by uncorrect dietary patterns [14]. Of note, current evidence concerning the relationship of some determinants (such as age, sex, education level, and marital status) and changes in consumption of MedDiet food components with the progressive shift in dietary habits is still controversial. For example, sex differences in MedDiet adherence were analyzed in several cohort-based studies, reporting higher [15,16] or lower [17] adherence to MedDiet in females compared to males. Instead, in a longitudinal study conducted in Northern Italy, reporting a low prevalence of MedDiet consumption that remained unchangedfrom 2010 to 2016, gender was not associated to MedDiet adherence, even though differences in counsumption of typical MedDiet food were reported [18].

Age has been also extensively investigated as a potential determinant of adherence to MedDiet. A cross-sectional analysis carried out on 3145 adults from 7 different countries reported a slightly higher MedDiet adherence by increasing age [19].

In a Croatian cohort, cross-sectional analyses found lower MedDiet adherence among younger compared to older, with a difference attributable to reduced consumption of wine, fish and seafood, and olive oil [20]. Age-dependent MedDiet adherence was also confirmed in Southern Italy [21] and Saudi Arabia [22].

In support of these cohort-based studies, evidence from a systematic literature review, including a wide number of studies from different world countries (Mediterranean, Middle Eastern and North African countries), reports an overall decrease in MedDiet adherence with no clear differences in MedDiet adherence by sex and age [23]. In an attempt to better identify changes in individual component consumption related to adherence to the MedDiet, we conducted a prospective study in a well characterized cohort from the PLIC study (Progression of Intimal Atherosclerotic Lesions in Carotid arteries [24]).

## 2. Materials and Methods

### 2.1. Study Population

Participants were selected among the subjects enrolled in the PLIC study, an ongoing single-centre, observational, cross-sectional, and longitudinal study of subjects enrolled on a voluntary basis in 1998 to 2000 and followed up for about 20 years (to date a total of 6 visits, on average every 4 years [24]). The study is conducted by the Center for the Study of Atherosclerosis at the E. Bassini Hospital (Cinisello Balsamo, Milan, Italy) with the coordination of the Epidemiology and Preventive Pharmacology Centre (SEFAP) of the Università degli Studi di Milano (Milan, Italy). The study was approved by the Scientific Committee of the Università degli Studi di Milano (SEFAP/Pr.0003). An informed consent was obtained by subjects, in accordance with the Declaration of Helsinki.

### 2.2. Biochemistry and Clinical Parameters

Subjects enrolled in the PLIC study undergo periodic visits to collect data on clinical parameters, patient-reported personal and familial pathological history, lifestyle habits, and drug therapies, together with blood samples to measure lipid and glycaemic profiles, as described elsewhere [25]. The prevalence of metabolic syndrome (MetS) was defined as the coexistence of at least three factors: triglyceride levels ≥ 150 mg/dL (or taking drug treatments for elevated triglycerides as an alternate indicator), blood pressure ≥ 130/85 mmHg, fasting glucose ≥ 100 mg/dL, reduced HDL-C (<40 mg/dL for men and <50 mg/dL for women), or having elevated waist circumference (≥102/88 cm [European recommended threshold] for men and women, respectively [26]).

### 2.3. The MEDAS Score

Our analysis used data from the MedDiet adherence (MEDAS) score [27], administered to PLIC study subjects during visits 5 (2017–2018) and 6 (2021–2022). The 14-item MEDAS tool was developed in a Spanish case-control study evaluating the impact of MedDiet on myocardial infarction incidence (the PREDIMED study [28,29]), where the best cut-offs for discriminating between cases and controls were established for each food or food group. MEDAS components are detailed in Table 1. The baseline 14-item questionnaire tabtabwas used to appraise adherence of participants to the MedDiet. Optimal adherence to MedDiet was considered for MEDAS score values of 8 or higher. It was chosen to consider a value of 8 or greater as an indicator of high/optimal adherence to MedDiet after considering previous studies and the related cut-offs [30,31].

### 2.4. Statistical Analysis

For the purpose of this investigation, among the subjects recruited for the PLIC study, we selected only those who underwent visit 5 (here considered as baseline visit), in which the MEDAS was introduced, and visit 6 (here considered as follow-up visit). Then, the absolute change in the score between follow-up and baseline visit was calculated for each individual (ΔMEDAS = MEDAS follow-up- MEDAS baseline), in order to categorize these subjects according to the change in the score (worsened, improved, or remained the same). We also evaluated the variation in the proportion of patients reporting to meet each MEDAS criteria, using the same approach (for example, for ‘Q1. Do you use olive oil as main culinary fat?’, the percentage variation was calculated as Δ%Q1 = [Proportion of subject reporting ‘Yes’ to Q1_follow-up_ − Proportion of subject reporting ‘Yes’ to Q1_baseline_]/Proportion of subject reporting ‘Yes’ to Q1_follow-up_).

Continuous variables were tested for normality using the Kolmogorov-Smirnov test. Data are presented as medians and 25th and 75th percentiles for continuous variables with a non-normal distribution or means ± standard deviations (SD) for variables with a normal distribution. Categorical variables are reported as counts and percentages. Differences between cohorts were analyzed using the non-parametric Mann-Whitney test or Student’s parametric *t*-test for continuous variables (or paired *t*-test for comparisons between baseline and follow-up visits) and the chi-square test or Fisher’s exact test for categorical variables.

Stratified analyses by age groups (≤50, 50–65, 65–80, >80 years [yrs], defined at baseline visit), sex, and change of MEDAS score (worsened, improved, unchanged) were also performed. Statistical significance was set at the 0.05 level for every analysis performed. Statistical analysis was performed using SAS (Statistical Analysis System) software version 9.4 (SAS. Institute, Inc., Cary, NC, USA).

## 3. Results

Of 1354 subjects of the whole PLIC cohort alive at the baseline examination, the complete information necessary for the analyses was available for 711 subjects. The mean age at the baseline visit was 68.1 ± 10.0 years, and about 58% of the participants were women. Other characteristics of the study population are reported in Supplementary Table S1, for both baseline and follow-up visits.

At baseline, the mean MEDAS score was 8.72 ± 1.82, with no appreciable differences by sex and age groups, and about 75% of the cohort presented optimal adherence to MedDiet (Table 2).

After 4.5 years of follow-up, on average, the mean MEDAS score was slightly lower (8.38 ± 1.4), but statistically different (*p*-value < 0.0001). In general, a reduction in the score was observed in both sexes and in all age groups, but it was greater and significant only for women (from 8.72 to 8.25, −5%;from 8.73 to 8.56, −2% for men) and for those aged 50–65 years (from 8.49 to 8.06, −5%, compared to a range of −2% and −4% in the other age groups, Table 2), although not significant.

We observed that about 34% of the sample improved their adherence to MedDiet (ΔMEDAS > 0), while 48% of the subjects worsened adherence (ΔMEDAS < 0). Among subjects who increased their adherence at follow-up, the score improvement averaged about two points (mean ΔMEDAS: +1.87 ± 1.13), as for those who worsened their adherence level (mean ΔMEDAS: −2.02 ± 1.14).

Overall, only three items showed a percentage increase: Q14 (dishes seasoned with sofrito at least two times per week, +48%), Q11 (not more than two portions of commercial sweets or pastries, +19%), and Q6 (no more than one serving of butter, margarine, or cream per day, +3%) (Supplementary Figure S1). These variations did not show significant differences across sexes and age groups (Supplementary Figures S2 and S3). 

The items with the higher percentage decrease were Q4 (at least three portions of fruit per day, −86%) and Q2 (at least 4 tablespoons of olive oil per day, −82%) (Supplementary Figure S1). While in the former case no relevant variations were observed in the two sexes and between age groups, for the latter there were differences in the percentage variation in the two sexes (−60% in women vs. −37% in men) and between age groups (−60% in subjects 65–80 years old, −29% in subjects 51–65 years old, and −20% in subjects ≤50 or >80 years old). Regarding sex, another item showing difference in percentage variation was Q12 (at least three servings of nuts per week: −45% women, −24% men). Finally, regarding age groups, the pattern was different for Q12 (at least three servings of nuts per week: +17% in subjects ≤50 years old vs. decreases [between −40% and −44%] in the other age groups) and for Q13 (preferential consumption of chicken, turkey, or rabbit meat instead of veal, pork, hamburger, or sausage: −8% in subjects ≤50 years old vs. increases [between +3% and +10%] in the other age groups).

**Figure 1:**
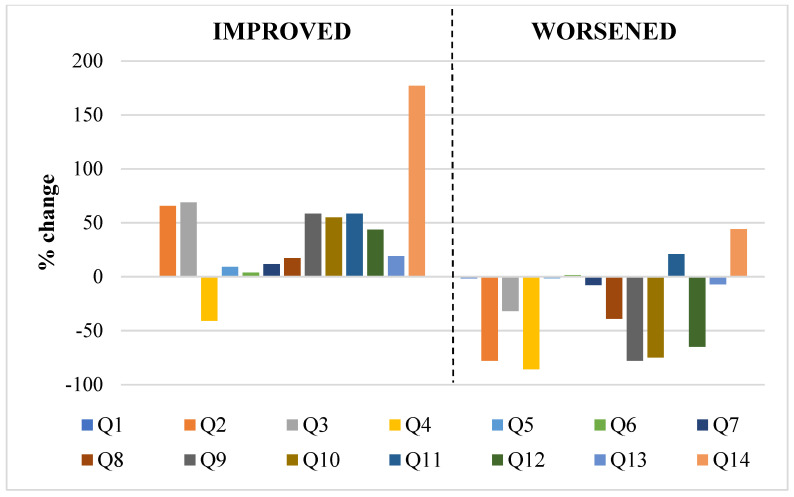
Percentage change from baseline of each item (Q; Table 1) assessed in the PREDIMED score.

Food items that showed the greatest variations in subjects who increased their MEDAS score were (Figure 1): olive oil (+66%, Q2), legumes (+59%, Q9) and fish consumption (+55%, Q10) and the use of dishes seasoned with sofrito (+177%, Q14). Within this subgroup that increased the MEDAS score, all items showed an improvement, with the exception of fruit consumption (−41%, Q4).

Instead, items that showed the greater variation in subjects in which MEDAS score decreased were (Figure 1): fruit (−86%, Q4), olive oil (−78%, Q2), legumes (−77%, Q9), fish (−75%, Q10) and nuts (−65%, Q12) consumption. Within this subgroup that decreased the MEDAS score, all items showed a worsening, with the exception of commercial sweets or pastries (+21%, Q11) and dishes seasoned with sofrito (+44%, Q14).

No significant differences in age, sex, or educational level emerged between subjects who increased their MedDiet adherence and those who decreased it (Table 3). Subjects who presented an improvement in the score during time showed higher body weight (73.55 ± 14.83 vs. 71.15 ± 13.68 kg, *p*-value = 0.04) and BMI (27.97 ± 4.85 vs. 27.17 ± 4.25 kg/m^2^, *p*-value = 0.04), higher glucose levels (107.39 ± 60.76 vs. 103.9 ± 19.25 mg/dL, not significant), but lower blood pressure (SBP 109.64 ± 32.24 vs. 116.81 ± 50.42 mmHg, *p*-value = 0.04; DBP 66.88 ± 19.21 vs. 69.64 ± 18.06 mmHg, *p*-value = 0.08) at baseline, compared with subjects who worsened their MedDiet adherence.

## 4. Discussion

Adherence to MedDiet is believed to promote health in the mid and long-term [32]. As a consequence, poor dietary habit appears as a modifiable risk factor for one of the most prevalent and impactful chronic diseases on health worldwide, which are cardiovascular diseases. A systematic review in adults evaluated the association between dietary factors including intake of vegetables, nuts, monounsaturated fatty acids, foods with a high glycaemic index, trans–fatty acids, and overall diet quality and dietary patterns, and concluded that a Mediterranean dietary pattern is causally protective against coronary heart disease [33].

In the present study, we assessed the change in adherence to MedDiet over a mid-term follow-up (4.5 yrs) in a cohort of free-living subjects, located in an urban area of Milan, in Northern Italy. In this cohort, adherence to MedDiet at baseline was 8.72, with a slight decrease after the follow-up period to 8.38; this reduction in MedDiet adherence was reflected by almost half of the study cohort (48%) that worsened its MEDAS score, with higher rates of worsening in women and in subjects aged 50–65 years.

Other studies conducted in Italy have reported conflicting results. A study by Pelucchi et al. on 3247 adults (mean age 56.4 years) from the Milan area showed that adherence to the MedDiet did not significantly change between 1991 and 2006, with the exception of a slight decrease in vegetables and meat intake [17]. A study conducted in the population of Southern Italy comparing diet habits between 1985–1986 to 2005–2006 reported a sharp decline in adherence to MedDiet [34], confirmed also by another analysis of a Southern Italy cohort from 2005 to 2010 [35], while a study investigating food consumption trends from 2010 to 2016 in subjects living in Northern Italy showed that overall prevalence of adherence to MedDiet remained constantly low, although with a marked increase in nuts consumption, a slight increase in white meat consumption, and a decrease in the consumption of fruit, red meat, sweets and sugar-sweetened beverages and in the use of sofrito [18].

This evidence suggests that dietary patterns depend on the period of analysis, geographical context [10], and population characteristics. We found age and sex-related variations. In particular, women and middle-old subjects (65–80 years) showed the highest variation in consuming at least 4 tablespoons of olive oil per day. Instead, men and subjects aged >50 years were the ones with the higher variations in consuming not more than two portions of commercial sweets or pastries and more than 3 servings of nuts per week, respectively. Age emerged as a determining factor, and in the studies conducted in Southern Italy presented above, the young population was the one mainly responsible for the reduction in adherence. Conversely, in our cohort, the 65–80 yrs age group was the one with the greatest reduction in olive oil and fruit consumption. However, these studies differ not only for the geographical setting, but also for study period, being the PLIC study performed about 10–15 years later than this age-frame. In this specific case, it is very difficult to discriminate whether the dietary pattern changes due to cohort ageing or to secular trends.

Overall, fruit consumption appears to be clearly declining (−86% of those who report consuming at least 3 portions per day), as well as oil consumption (−82% of those who report consuming at least 4 tablespoons per day). The recent economic crisis in Europe has been proposed to have a role, due to an increase in the prices of some food items typical of the Mediterranean diet pyramid, such as fruits, compared to the more affordable prices of refined grains, sweets and snacks [36,37]. Of note, we investigated variations in MedDiet adherence during a particular timeframe, heavily affected by forced lifestyle changes, social distancing and isolation at home due to COVID-19 pandemic restrictions, which also impacted dietary intakes [38,39].

While recognizing an overall worsening of adherence to MedDiet, our study found that one third of the sample showed an improvement. This change was mainly driven by an increase in the proportion of subjects that use at least two dishes seasoned with sofrito per week (+48%). The other two main driving items were the consumption of at least 4 tablespoons of oil per day and of at least two vegetable servings per day. Interestingly, these items were also highly decreased among the subgroup of subjects who showed a decrease in MEDAS score. This basically indicated that some items in the score are more subject to variation over time, and more sensitive to changes in the social environment (e.g., economic conditions) or in the daily habits of the population. These items are likely to be the primary targets of intervention, whether educational or economical.

We did not find significant differences in terms of mean age and gender between the subsample showing a worsening and the subsample showing an improvement in MEDAS score. However, the subgroup with increased MEDAS score showed higher body weight and BMI, as also higher glucose levels (although not significant) compared to the subgroup with decreased MEDAS score, suggesting that the increasing adherence to MedDiet may be secondary to the recognition of a compromised metabolic situation, which could be improved by lifestyle modifications.

Our study has some limitations. First, our results may not be fullt representative to the whole general population, since reflect changes in MedDiet adherence in a middle-aged cohort living in an urban setting in Northern Italy. However, the PLIC study is still ongoing, and longer-term data in this cohort, as well as in a younger group, will allow to better describe the actual changes in MedDiet adherence and their impact on cardiovascular and metabolic health. Second, other possible determinants of dietary quality might have not been evaluated in this study. Third, another limitation is the recall-bias due to the conduct of the investigation related to visit 6 during a difficult period that could have generated alterations in the perceptions of participants compared to the period prior to the COVID-19 lockdown.

## 5. Conclusions

Our results suggest a trend toward a worsening of dietary habits, albeit with small changes, evaluated during a timeframe heavily affected by COVID-19 pandemic. The observed lowering of MedDiet adherence was driven by an overall reduction in fruit and olive oil. Specific items of the score were shown to be more variable, both among sexes and age classes. These results open up the possibility to design more tailored dietary intervention based on individual features to target specific food items for MedDiet adherence improvement.

## Figures and Tables

**Table 1:** Validated 14-item questionnaire of Mediterranean diet adherence.

Questions	Criteria for 1 Point
1. Do you use olive oil as main culinary fat?	Yes
2. How much olive oil do you consume in a given day (including oil used for frying, salads, out-of-house meals, etc.)?	≥4 tablespoons
3. How many vegetable servings do you consume per day? (1 serving: 200 g [consider side dishes as half a serving])	≥2 (≥1 portion raw or as a salad)
4. How many fruit units (including natural fruit juices) do you consume per day?	≥3
5. How many servings of red meat, hamburger, or meat products (ham, sausage, etc.) do you consume per day? (1 serving: 100–150 g)	<1
6. How many servings of butter, margarine, or cream do you consume per day? (1 serving: 12 g)	<1
7. How many sweet or carbonated beverages do you drink per day?	<1
8. How much wine do you drink per week?	≥7 glasses
9. How many servings of legumes do you consume per week? (1 serving: 150 g)	≥3
10. How many servings of fish or shellfish do you consume per week? (1 serving 100–150 g of fish or 4–5 units or 200 g of shellfish)	≥3
11. How many times per week do you consume commercial sweets or pastries (not homemade), such as cakes, cookies, biscuits, or custard?	<3
12. How many servings of nuts (including peanuts) do you consume per week? (1 serving 30 g)	≥3
13. Do you preferentially consume chicken, turkey, or rabbit meat instead of veal, pork, hamburger, or sausage?	Yes
14. How many times per week do you consume vegetables, pasta, rice, or other dishes seasoned with sofrito (sauce made with tomato and onion, leek, or garlic and simmered with olive oil)?	≥2

**Table 2:** Mediterranean diet adherence at baseline and follow-up visits.

	Baseline Visit	Follow-Up Visit	*p*-Value
MEDAS score; mean (SD)	8.72 (1.82)	8.38 (1.45)	<0.0001
Optimal adherence to Mediterranean diet; %	74.82	72.86	<0.0001
MEDAS score by sex; mean (SD)			
Females (N = 414)	8.72 (1.75)	8.25 (1.42)	<0.0001
Males (N = 297)	8.73 (1.91)	8.56 (1.47)	0.17
MEDAS score by yrs age groups; mean (SD)			
≤50 (N = 47)	8.09 (2.09)	7.91 (1.41)	0.55
50–65 (N = 177)	8.49 (1.85)	8.06 (1.56)	0.31
65–80 (N = 451)	8.89 (1.78)	8.51 (1.40)	<0.0001
>80 (N = 36)	8.58 (1.42)	8.36 (1.46)	0.30
Classes of adherence to Mediterranean diet; %			
Improved (N = 243)		34.18	
Unchanged (N = 122)		17.16	
Worsened (N = 346)		48.66	

MEDAS, MedDiet adherence; SD, standard deviation.

**Table 3:** Comparison of demographic and clinical characteristics at the baseline visit by classes of adherence to Mediterranean diet.

Covariates	Improved Score N = 243 (34%)	Worsened Score N = 346 (48%)	*p*-Value
Age, years; mean (±SD)	67.6 (9.74)	68.53 (9.71)	0.25
Women, %	53.91	60.4	0.12
Educational level, %			
Primary schools	18.93	18.50	0.93
Secondary schools	34.16	34.97	
High schools	38.68	38.44	
University degree	7.41	7.80	
Weight, kg; mean (SD)	73.55 (14.83)	71.15 (13.68)	0.04
BMI, kg/m^2^; mean (SD)	27.97 (4.85)	27.17 (4.25)	0.04
WH ratio; mean (SD)	0.91 (0.08)	0.9 (0.09)	0.13
SBP, mm Hg; mean (SD)	109.64 (32.24)	116.81 (50.42)	0.04
DBP, mm Hg; mean (SD)	66.88 (19.21)	69.64 (18.06)	0.08
Total Chol, mg/dL; mean (SD)	197.61 (34.18)	194.72 (35.97)	0.33
HDL-C, mg/dL; mean (SD)	56.93 (13.81)	57.73 (13.76)	0.49
LDL-C, mg/dL; mean (SD)	118.36 (29.39)	114.54 (30.4)	0.13
TG, mg/dL; median (IQR)	98 (76–131)	94 (76–136)	0.89
Fasting glucose, mg/dL; mean (SD)	107.39 (60.76)	103.9 (19.25)	0.40
Dietary Therapy, %	8.64	8.38	0.91
Smoker, %	10.46	12.14	0.48
Physical activity, %	29.58	30.72	0.17
Antihypertensive treatment, %	16.46	21.68	0.12
Antidiabetic treatment, %	3.7	1.45	0.08
Lipid-lowering treatment, %	34.16	35.55	0.73
MetS, %	67.6 (9.74)	68.53 (9.71)	0.25
cIMT, mm; mean (SD)	0.79 (0.16)	0.80 (0.16)	0.75

BMI indicates body mass index; WH, waist-to-hip; SBP, systolic blood pressure; DBP, dystolic blood pressure; Total Chol, total cholesterol; HDL-C, high-density lipoprotein cholesterol; LDL-C, low-density lipoprotein cholesterol; TG, triglycerides; MetS, metabolic syndrome; cIMT, carotid intima-media thickness.

## Data Availability

The pooled data that support the findings of this study are available from the author A.L.C., upon reasonable request.

## Supplementary Materials

The following supporting information can be downloaded at: https://www.mdpi.com/article/10.3390/nu15081844/s1, Table S1: Demographic and clinical characteristics of participants; Figure S1: Percentage change from baseline of each item (Q) assessed in the MEDAS score for the entire cohort; Figure S2: Percentage change from baseline of each item (Q) assessed in the MEDAS score by sex; Figure S3: Percentage change from baseline of each item (Q) assessed in the MEDAS score by age groups.

## References

[1] Martínez-González M.A., Villegas A.S. (2003). The emerging role of Mediterranean diets in cardiovascular epidemiology: Monounsaturated fats, olive oil, red wine or the whole pattern?. Eur. J. Epidemiol..

[2] Guasch-Ferré M., Willett W.C. (2021). The Mediterranean diet and health: A comprehensive overview. J. Intern. Med..

[3] Bucciantini M., Leri M., Nardiello P., Casamenti F., Stefani M. (2021). Olive Polyphenols: Antioxidant and Anti-Inflammatory Properties. Antioxidants.

[4] Dominguez L., Di Bella G., Veronese N., Barbagallo M. (2021). Impact of Mediterranean Diet on Chronic Non-Communicable Diseases and Longevity. Nutrients.

[5] Trevisan M., Krogh V., Grioni S., Farinaro E. (2020). Mediterranean diet and all-cause mortality: A cohort of Italian men. Nutr. Metab. Cardiovasc. Dis..

[6] Hidalgo-Liberona N., Meroño T., Zamora-Ros R., Rabassa M., Semba R., Tanaka T., Bandinelli S., Ferrucci L., Andres-Lacueva C., Cherubini A. (2021). Adherence to the Mediterranean diet assessed by a novel dietary biomarker score and mortality in older adults: The InCHIANTI cohort study. BMC Med..

[7] Estruch R., Ros E., Salas-Salvadó J., Covas M.-I., Corella D., Arós F., Gómez-Gracia E., Ruiz-Gutiérrez V., Fiol M., Lapetra J. (2018). Retraction and Republication: Primary Prevention of Cardiovascular Disease with a Mediterranean Diet. N. Engl. J. Med..

[8] Eleftheriou D., Benetou V., Trichopoulou A., La Vecchia C., Bamia C. (2018). Mediterranean diet and its components in relation to all-cause mortality: Meta-analysis. Br. J. Nutr..

[9] Tang C., Wang X., Qin L.-Q., Dong J.-Y. (2021). Mediterranean Diet and Mortality in People with Cardiovascular Disease: A Meta-Analysis of Prospective Cohort Studies. Nutrients.

[10] Mattavelli E., Olmastroni E., Bonofiglio D., Catapano A.L., Baragetti A., Magni P. (2022). Adherence to the Mediterranean Diet: Impact of Geographical Location of the Observations. Nutrients.

[11] Visseren F.L.J., Mach F., Smulders Y.M., Carballo D., Koskinas K.C., Bäck M., Benetos A., Biffi A., Boavida J.-M., Capodanno D. (2022). 2021 ESC Guidelines on cardiovascular disease prevention in clinical practice. Eur. J. Prev. Cardiol..

[12] Quintana-Navarro G.M., Alcala-Diaz J.F., Lopez-Moreno J., Perez-Corral I., Leon-Acuña A., Torres-Peña J.D., Rangel-Zuñiga O.A., De Larriva A.P.A., Corina A., Camargo A. (2020). Long-term dietary adherence and changes in dietary intake in coronary patients after intervention with a Mediterranean diet or a low-fat diet: The CORDIOPREV randomized trial. Eur. J. Nutr..

[13] Lacirignola C., Capone R., El Bilali H., Debs P., Cardone G.L., Driouech N., Dernini S.B.B., Gitz V.M.A. (2017). Priority 5, Mediterranean food consumption patterns: Diet, environment, society, economy and health. Feed. Knowl..

[14] GBD 2019 Diseases and Injuries Collaborators (2020). Global burden of 369 diseases and injuries in 204 countries and territories, 1990–2019: A systematic analysis for the Global Burden of Disease Study 2019. Lancet.

[15] Adjibade M., Assmann K.E., Andreeva V.A., Lemogne C., Hercberg S., Galan P., Kesse-Guyot E. (2018). Prospective association between adherence to the Mediterranean diet and risk of depressive symptoms in the French SU.VI.MAX cohort. Eur. J. Nutr..

[16] Maraki M.I., Yannakoulia M., Stamelou M., Stefanis L., Xiromerisiou G., Kosmidis M.H., Dardiotis E., Hadjigeorgiou G.M., Sakka P., Anastasiou C.A. (2019). Mediterranean diet adherence is related to reduced probability of prodromal Parkinson’s disease. Mov. Disord..

[17] Pelucchi C., Galeone C., Negri E., La Vecchia C. (2010). Trends in adherence to the Mediterranean diet in an Italian population between 1991 and 2006. Eur. J. Clin. Nutr..

[18] Leone A., Battezzati A., Bertoli S., De Amicis R., De Carlo G. (2017). Trends of Adherence to the Mediterranean Dietary Pattern in Northern Italy from 2010 to 2016. Nutrients.

[19] Quarta S., Massaro M., Chervenkov M., Ivanova T., Dimitrova D., Jorge R., Andrade V., Philippou E., Zisimou C., Maksimova V. (2021). Persistent Moderate-to-Weak Mediterranean Diet Adherence and Low Scoring for Plant-Based Foods across Several Southern European Countries: Are We Overlooking the Mediterranean Diet Recommendations?. Nutrients.

[20] Gerić M., Matković K., Gajski G., Rumbak I., Štancl P., Karlić R., Bituh M. (2022). Adherence to Mediterranean Diet in Croatia: Lessons Learned Today for a Brighter Tomorrow. Nutrients.

[21] Grosso G., Marventano S., Giorgianni G., Raciti T., Galvano F., Mistretta A. (2014). Mediterranean diet adherence rates in Sicily, southern Italy. Public Health Nutr..

[22] Aljabri M.K., Al-Raddadi R., Bahijri S.M., Al Ahmadi J., Ajabnoor G., Jambi H.A. (2019). Factors associated with adherence to Mediterranean diet among Saudi non-diabetic patients attending primary health care centers: A cross-sectional study. J. Taibah Univ. Med. Sci..

[23] Obeid C.A., Gubbels J.S., Jaalouk D., Kremers S.P.J., Oenema A. (2022). Adherence to the Mediterranean diet among adults in Mediterranean countries: A systematic literature review. Eur. J. Nutr..

[24] Olmastroni E., Baragetti A., Casula M., Grigore L., Pellegatta F., Pirillo A., Tragni E., Catapano A.L. (2019). Multilevel Models to Estimate Carotid Intima-Media Thickness Curves for Individual Cardiovascular Risk Evaluation. Stroke.

[25] Olmastroni E., Shlyakhto E.V., Konradi A.O., Rotar O.P., Alieva A.S., Boyarinova M.A., Baragetti A., Grigore L., Pellegatta F., Tragni E. (2018). Epidemiology of cardiovascular risk factors in two population-based studies. Atheroscler. Suppl..

[26] Expert Panel on Detection, Evaluation, and Treatment of High Blood Cholesterol in Adults (2001). Executive Summary of The Third Report of The National Cholesterol Education Program (NCEP) Expert Panel on Detection, Evaluation, And Treatment of High Blood Cholesterol In Adults (Adult Treatment Panel III). JAMA.

[27] Schröder H., Fitó M., Estruch R., Martínez-González M.A., Corella D., Salas-Salvadó J., Lamuela-Raventós R., Ros E., Salaverría I., Fiol M. (2011). A Short Screener Is Valid for Assessing Mediterranean Diet Adherence among Older Spanish Men and Women. J. Nutr..

[28] Martínez-González M.A., Fernández-Jarne E., Serrano-Martínez M., Marti A., Martinez J.A., Martín-Moreno J.M. (2002). Mediterranean diet and reduction in the risk of a first acute myocardial infarction: An operational healthy dietary score. Eur. J. Nutr..

[29] Martinez-Gonzalez M.A., Fernández-Jarne E., Serrano-Martínez M., Wright M., Gomez-Gracia E. (2004). Development of a short dietary intake questionnaire for the quantitative estimation of adherence to a cardioprotective Mediterranean diet. Eur. J. Clin. Nutr..

[30] Sánchez-Taínta A., Estruch R., Bulló M., Corella D., Gómez-Gracia E., Fiol M., Algorta J., Covas M.-I., Lapetra J., Zazpe I. (2008). Adherence to a Mediterranean-type diet and reduced prevalence of clustered cardiovascular risk factors in a cohort of 3204 high-risk patients. Eur. J. Cardiovasc. Prev. Rehabil..

[31] León-Muñoz L.M., Guallar-Castillón P., Graciani A., López-García E., Mesas A.E., Aguilera M.T., Banegas J.R., Rodríguez-Artalejo F. (2012). Adherence to the Mediterranean Diet Pattern Has Declined in Spanish Adults. J. Nutr..

[32] Afshin A., Sur P.J., Fay K.A., Cornaby L., Ferrara G., Salama J.S., Mullany E.C., Abate K.H., Abbafati C., Abebe Z. (2019). Health effects of dietary risks in 195 countries, 1990–2017: A systematic analysis for the Global Burden of Disease Study 2017. Lancet.

[33] Mente A., De Koning L., Shannon H.S., Anand S.S. (2009). A Systematic Review of the Evidence Supporting a Causal Link Between Dietary Factors and Coronary Heart Disease. Arch. Intern. Med..

[34] Veronese N., Notarnicola M., Cisternino A.M., Inguaggiato R., Guerra V., Reddavide R., Donghia R., Rotolo O., Zinzi I., Leandro G. (2020). Trends in adherence to the Mediterranean diet in South Italy: A cross sectional study. Nutr. Metab. Cardiovasc. Dis..

[35] Bonaccio M., Di Castelnuovo A., Bonanni A., Costanzo S., De Lucia F., Persichillo M., Zito F., Donati M.B., de Gaetano G., Iacoviello L. (2014). Decline of the Mediterranean diet at a time of economic crisis. Results from the Moli-sani study. Nutr. Metab. Cardiovasc. Dis..

[36] Sikorski C., Yang S., Stennett R., Miller V., Teo K., Anand S.S., Paré G., Yusuf S., Dehghan M., Mente A. (2022). Changes in energy, macronutrient, and food consumption in 47 countries over the last 70 years (1950–2019): A systematic review and meta-analysis. Nutrition.

[37] Bonaccio M., Bes-Rastrollo M., de Gaetano G., Iacoviello L. (2016). Challenges to the Mediterranean diet at a time of economic crisis. Nutr. Metab. Cardiovasc. Dis..

[38] O’Connell M., Smith K., Stroud R. (2022). The dietary impact of the COVID-19 pandemic. J. Health Econ..

[39] Celorio-Sardà R., Comas-Basté O., Latorre-Moratalla M.L., Zerón-Rugerio M.F., Urpi-Sarda M., Illán-Villanueva M., Farran-Codina A., Izquierdo-Pulido M., Vidal-Carou M.D.C. (2021). Effect of COVID-19 Lockdown on Dietary Habits and Lifestyle of Food Science Students and Professionals from Spain. Nutrients.

