# Supplementary material for: Adherence to Mediterranean Diet: A Population-Based Longitudinal Cohort Study
Source: Nutrients. 2023 Apr 12;15(8):1844. doi: 10.3390/nu15081844 (PMC10145158; doi:10.3390/nu15081844)

SUPPLEMENTARY MATERIAL

ADHERENCE TO MEDITERRANEAN DIET IN OLDER SUBJECTS: A POPULATION-BASED MID-TERM LONGITUDINAL COHORT STUDY

Authors

Elisa Mattavelli [1,2], Elena Olmastroni [2,3], Manuela Casula [2,3], Liliana Grigore [2], Fabio Pellegatta [2], Andrea Baragetti[1,2], Paolo Magni[1,2] , Alberico L. Catapano[2]

Affiliations

1 Department of Pharmacological and Biomolecular Sciences, Università degli Studi di Milano, Milan 20133, Italy; elisa.mattavelli@unimi.it (E.M.); andrea.baragetti@unimi.it (A.B.); paolo.magni@unimi.it (P.M.);

2 MultiMedica IRCCS, 20092 Cinisello Balsamo, Milan, Italy; grigore.centroatero@gmail.com (L.G.); fabio.pellegatta@guest.unimi.it (F.P.); alberico.catapano@multimedica.it (A.L.C);

3 Epidemiology and Preventive Pharmacology Service (SEFAP), Department of Pharmacological and Biomolecular Sciences, Università degli Studi di Milano, 20133 Milan, Italy; elena.olmastroni@unimi.it (E.O.); manuela.casula@unimi.it (M.C.).

Corresponding author

Paolo Magni

Department of Pharmacological and Biomolecular Sciences, Universita' degli Studi di Milano, 20133, Milan, Italy; IRCCS MultiMedica, Sesto San Giovanni, 20099, Milan, Italy.

Electronic address: paolo.magni@unimi.it.

Keywords: Mediterranean diet, adherence, cardiovascular disease, metabolic disease

Supplementary Table S1. Demographic and clinical characteristics of participants.

**Covariates**	**Baseline visit**	**Follow-up visit**	**p-value**
Weight, kg; mean (SD)	71.81 (14.03)	71.24 (14)	0.45
BMI, kg/m^2; mean (SD)	27.41 (4.5)	27.6 (4.62)	0.43
WH ratio; mean (SD)	0.9 (0.09)	0.93 (0.09)	<.0001
SBP, mm Hg; mean (SD)	113.7 (42.18)	126.95 (17.02)	<.0001
DBP, mm Hg; mean (SD)	68.48 (18.57)	75.33 (10.62)	<.0001
Total Chol, mg/dL; mean (SD)	196.42 (35.81)	184.45 (34.58)	<.0001
HDL-C, mg/dL; mean (SD)	57.65 (13.93)	55.58 (15.11)	0.01
LDL-C, mg/dL; mean (SD)	116.36 (30.69)	107.37 (29.91)	<.0001
TG, mg/dL; median [IQR]	97 [76-134]	101 [76-126]	0.63
Fasting glucose, mg/dL; mean (SD)	105.16 (39.16)	100.05 (17.17)	0.002
Dietary Therapy, %	8.3	7.45	0.55
Smoker, %	11.88	10.22	0.57
Physical activity, %	50.07	46.12	<.0001
Antihypertensive treatment, %	19.27	34.46	<.0001
Antidiabetic treatment, %	2.67	8.16	<.0001
Lipid-lowering treatment, %	35.02	23.49	<.0001
MetS, %	27.99	35.32	0.003
cIMT, mm; mean (SD)	0.79 (0.16)	0.82 (0.14)	<.0001

BMI indicates body mass index; WH, waist-to-hip; SBP, systolic blood pressure; DBP, dystolic blood pressure; Total Chol, total cholesterol; HDL-C, high-density lipoprotein cholesterol; LDL-C, low-density lipoprotein cholesterol; TG, triglycerides; MetS metabolic syndrome; cIMT, carotid intima-media thickness;

Supplementary Figure S1. Percentage change from baseline of each item (Q) assessed in the MEDAS score for the entire cohort.

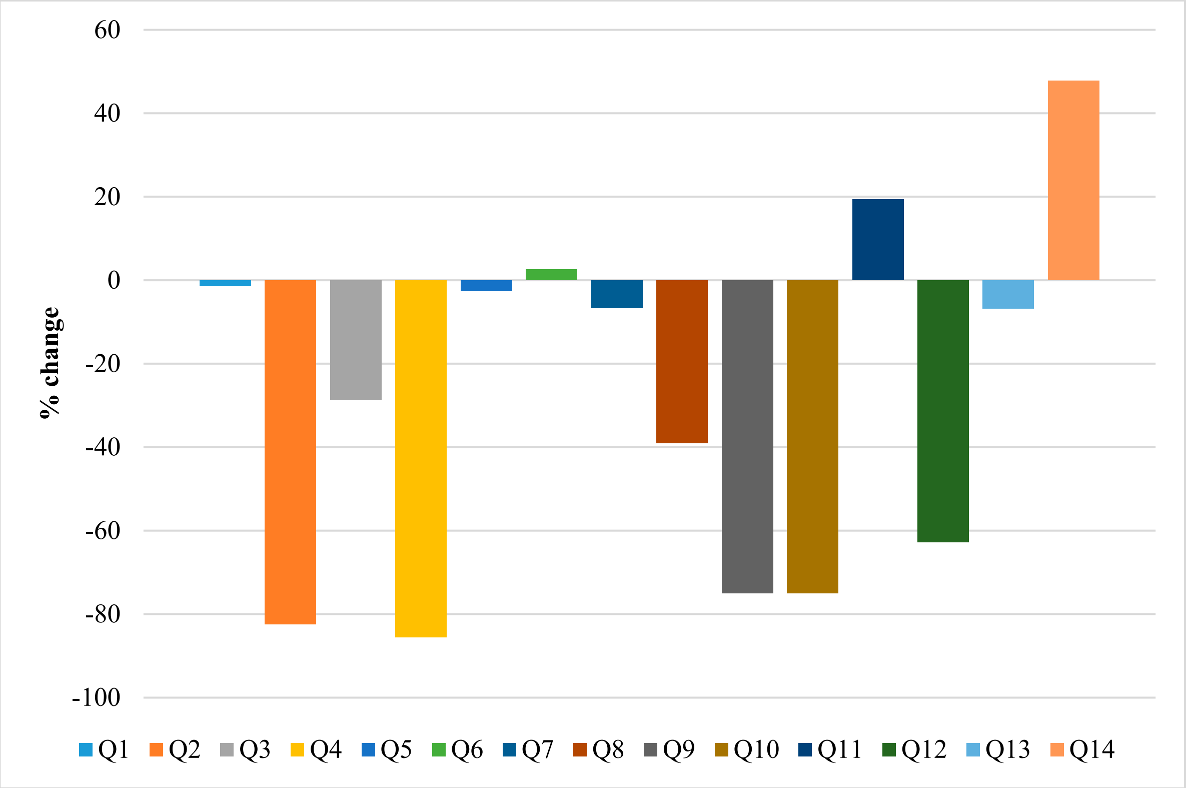

Supplementary Figure S2. Percentage change from baseline of each item (Q) assessed in the MEDAS score by sex.

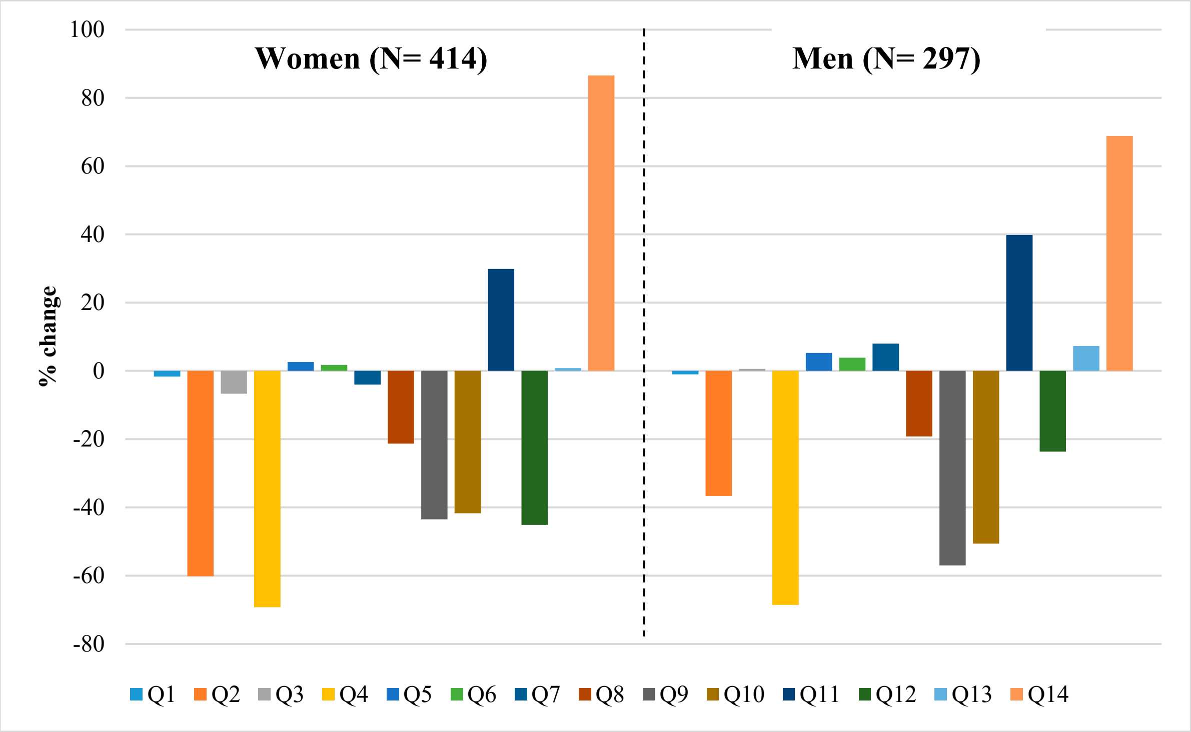

Supplementary Figure S3. Percentage change from baseline of each item (Q) assessed in the MEDAS score by age groups.

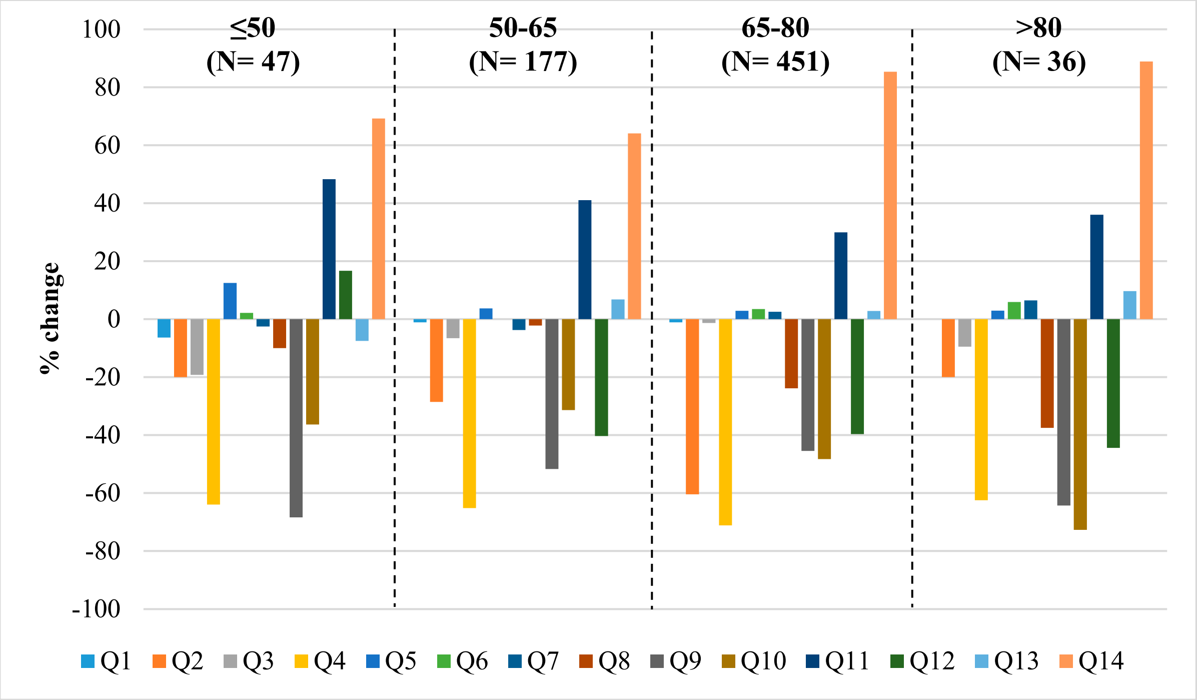

Supplement: Supplementary file 1 [file nutrients-15-01844-s001.zip › nutrients-2313790-supplementary.pdf]
